# The Efficacy of Yeast Phagostimulant Baits in Attract-and-Kill Strategies Varies between Summer- and Winter-Morphs of *Drosophila suzukii*

**DOI:** 10.3390/insects13110995

**Published:** 2022-10-29

**Authors:** Rory Jones, Paul E. Eady, Matthew R. Goddard, Michelle T. Fountain

**Affiliations:** 1 School of Life Sciences, University of Lincoln, Lincoln LN6 7DL, UK; 2 NIAB, East Malling, Kent ME19 6BJ, UK

**Keywords:** spotted wing drosophila, SWD, drosophilae, integrated pest management, pesticide, lure, toxic bait

## Abstract

**Simple Summary:**

The winter-morph of the soft and stone fruit pest *Drosophila suzukii* (Matsumura), commonly called spotted wing drosophila, differs in comparison to the summer-morph in terms of its response to olfactory cues. *D. suzukii* is predominantly controlled using conventional insecticide applications but this is not sustainable due to emerging insecticide resistance and the withdrawal of active ingredients. Combining phagostimulant baits with insecticides can significantly reduce the amount of insecticide used whilst maintaining levels of control. Yeasts are effective phagostimulants and in combination with insecticides may control *D. suzukii*, but only a limited number of single yeast species have been tested. We investigated the effectiveness of single and combinations of co-fermented yeasts combined with insecticides in laboratory assays and evaluated their effectiveness as phagostimulant baits for use in Integrated Pest Management strategies to control *D. suzukii*. This study found that some combinations of yeasts were more effective baits that single yeasts, and that certain yeasts were more effective phagostimulants for winter- than summer-morph *D. suzukii*. These findings suggest that yeast phagostimulants in attract-and-kill strategies should be adjusted to target summer- and winter-morph *D. suzukii* for more effective control.

**Abstract:**

*Drosophila suzukii* (Matsumura), is a globally invasive pest of soft and stone fruit. To survive winter in temperate zones it enters a reproductive diapause in a morphologically distinct phenotype. Phagostimulant baits can be combined with insecticides in attract-and-kill strategies for control. We investigated the effectiveness of single yeast species and combinations of co-fermented yeast phagostimulant baits when combined with insecticides in laboratory assays against both summer- and winter-morph *D. suzukii*. *Candida zemplininia* or *Hanseniaspora uvarum* + *C. zemplininia* combined with lambda-cyhalothrin or cyantraniliprole, and *H. uvarum* combined with cyantraniliprole caused significantly higher mortality in winter- compared to summer-morph *D. suzukii*. Additionally, lambda-cyhalothrin combined with *M. pulcherrima* + *H. uvarum* resulted in greater mortality compared to single yeasts, *H. uvarum* for both summer- and winter-morphs and *C. zemplininia* for summer-morphs. *M. pulcherrima* + *H. uvarum* with spinosad significantly reduced the time-to-kill (50%) of summer-morphs compared to insecticide alone. Most yeast-based baits were comparable in terms of attract-and-kill efficacy to Combi-protec, a commercially available bait, although *M. pulcherrima* or *H. uvarum* + *C. zemplininia* in with cyantraniliprole were less effective. Our study suggests that yeast phagostimulants in attract-and-kill strategies should be adjusted for summer- and winter-morph *D. suzukii* for more effective control.

## 1. Introduction

*Drosophila suzukii* (Matsumura) is a pest of soft and stone fruits that has spread from Southeast Asia being first identified invading fruit in the USA and Europe in 2008, then the UK in 2012 [1,2,3]. *D. suzukii*, unlike most other *Drosophila* species, can oviposit in ripening fruit [4] and is a major economic pest with annual losses to the soft fruit industry in Trento, Italy, estimated to be EUR 2.73 million [5].

Adult *D. suzukii* exhibit reproductive diapause which aids survival during unfavourable winter conditions. The winter phenotype is morphologically distinct, being larger and darker in colour than the summer-morph counterpart [6] and is associated with a longer lifespan at lower temperatures than the summer-morph [7]. Temperature is the main driving factor facilitating the transition between morphs [8]. During the latter stages of the growing season there was an increase in the prevalence of the winter-morph phenotype from ~30% at the end of September to ~99% by the end of December in the Netherlands [9]. Increases in winter-morph phenotypes were accompanied by the dispersion of *D. suzukii* into woodlands and hedgerows where they likely overwinter in sheltered microclimates such as crevices under loose bark or leaf litter [10,11,12]. Controlling winter-morph *D. suzukii* is key to reducing early fruit damage as winter-morph females make up the majority of the population entering commercial fruit crops in the spring [9].

*Drosophila suzukii* is predominantly controlled using management programmes containing conventional insecticide applications of various active ingredients [13,14]. Although, attractant-based traps for monitoring and control, crop hygiene, reduced harvest intervals, exclusion netting and pruning are all important for integrated pest management of *D. suzukii* [15,16,17]. Additionality, natural products and biological control also show promise for control of this pest [13,18]. Due to the withdrawal of several active ingredients and emerging insecticide resistance [19,20], reliance on chemical insecticides for control is not sustainable. Therefore, new control measures need to be developed and existing ones improved.

One attract-and-kill strategy which shows promise for use in Integrated Pest Management of *D. suzukii* is combining insecticides with phagostimulants, applying as narrow band or full foliar coverage sprays, to attract flies to a toxic food source [21,22,23,24,25,26,27,28]. Combining insecticides with phagostimulants increases the exposure of target insects to insecticides via the initial attraction to a bait, followed by increased cuticular contact with the toxic substance and through stimulation of feeding on the insecticide. Additionally, phagostimulants could increase the efficiency of less effective insecticides classes [23]. Attracting *D. suzukii* to feed on bait-insecticide combinations could limit the exposure of non-target organisms to insecticides whilst significantly reducing the dose and amount of insecticide by up to 96% while retaining comparable levels of control to conventional insecticide application [26], including reducing insecticide residues in fruit [28].

There is some evidence that olfactory attraction to baits varies between the winter and summer phenotypes [29,30]. In two-way laboratory choice tests summer-morph *D. suzukii* females were more attracted to strawberry juice compared to apple cider vinegar whilst the opposite was observed for winter-morph females [29]. Geosmin (a sesquiterpene with a distinct earthy odour) was shown to repel summer-morph flies whilst having a mildly positive chemotactic effect on winter-morph flies [30]. This may reflect differences in life-history traits associated with resource acquisition as it has been suggested that winter-morph flies are more opportunistic, feeding on decomposing vegetation [31]. In terms of chemotaxis towards (or away from) yeast-based baits, studies have shown that *Saccharomyces cerevisiae* (baker’s yeast) elicited no difference in attraction between *D. suzukii* morphs [30,32]. However, winter-morphs were more attracted to *Candida zemplinina* alone or when combined with *Hanseniaspora uvarum* [33]. Variance in olfactory attraction between morphs has important implications for attract-and-kill strategies, suggesting differential attractants may be required for the two morphs to optimise the performance of the baits.

Yeasts represent an important class of phagostimulants in the control of *D. suzukii* [22,24,26,27]. Several yeasts have been identified as being attractive to *D. suzukii* including *H. uvarum*, *H. opuntiae, C. zemplinina*, *C. californica, Pichia terricola*, *P. pijperi, Metschnikowia pulcherrima* and *S. cerevisiae* [24,34,35,36,37,38]. Combinations of *C. zemplinina*, *P. pijperi*, *P. terricola*, *M. pulcherrima* and *H. uvarum* are also attractive, although not significantly more attractive than *H. uvarum* alone [36,38]. However, the yeast combinations tested for attraction thus far have been simplistic, comprising of few species and/or were singly fermented then combined [36,38]. Naturally occurring yeast communities on *D. suzukii* fruit hosts are complex [39,40,41,42] and likely interact on the surface of fruit. This potential interaction may modulate attraction as ferments with *S. cerevisiae* and *Pichia kluyveri* produced synergistic metabolic interactions in terms of volatiles [43]. Further, there is evidence that attraction of *D. melanogaster* to co-cultures of *S. cerevisiae* and certain species of bacteria was enhanced compared to post-growth blending [44]. However, co-fermenting certain yeasts did not improve attraction [33]. In addition, reducing the number of non-target *Drosophila* species killed may be important to maximise inter-species competition [45,46] and yeasts could provide selective baits which discriminate between *Drosophila* species [36].

Various baits have been assessed for their effectiveness as phagostimulants for *D. suzukii*, including commercial products (mainly protein-based) and sugar and yeast, both separately and in combination. Combi-protec (Dedetec), a commercially available protein-based bait, both improved mortality and reduced egg laying of *D. suzukii* when combined with several different insecticides [24,26,28,47,48,49]. Adding brown cane sugar solution to spinosad or cyantraniliprole significantly increased the mortality of adult *D. suzukii* during laboratory assays [23,50]. Despite the identification of a range of yeast species that are attractive to *D. suzukii*, relatively few species have been assessed for their effectiveness as phagostimulants. *Saccharomyces cerevisiae* combined with sugar in combination with certain insecticides, spinosad but not cyantraniliprole, significantly increased *D. suzukii* mortality [23]. Additionally, *S. cerevisiae* and sugar baits combined with spinosad also increased mortality compared to commercially available protein-based baits (NuLure and GF-120) [50]. The effect of yeast phagostimulants may not be clear-cut as another study shows combining *S. cerevisiae* with spinosad and spinetoram lowered efficacy compared to insecticide alone after 8 h exposure, with equivalent efficacy after 16 h [51]. Factors such as host fruit availability and physiological state of *D. suzukii* may modulate the effectiveness of phagostimulants [51,52] potentially contributing to observed variation in success of these baits. Although *S. cerevisiae* is an effective phagostimulant bait, it is less attractive to *D. suzukii* than other yeast species like *H. uvarum* [24,34,36,38]. When combined with spinosad, cyantraniliprole or lambda-cyhalothrin, *H. uvarum* increased mortality and reduced oviposition (with lambda-cyhalothrin) compared to insecticide only controls and *H. uvarum* combined with spinosad was persistent on leaves and effective one week after application on grape leaves [25]. Spinosad and cyantraniliprole combined with *H. uvarum* also increased mortality of winter-morph *D. suzukii* compared to an insecticide only control and cyantraniliprole reduced oviposition of acclimatised winter-morph females [24]. Insecticides, both combined with phagostimulant baits and alone, were more effective against summer- than winter-morphs [24]. However, summer-morphs were maintained at a higher temperature with longer light conditions likely affecting *D. suzukii* activity and hence, it is not clear how this influenced mortality [24].

Increased attractiveness of phagostimulants baits may not result in increased effectiveness when combined with insecticides as Combi-protec was significantly less attractive than *H. uvarum* to summer-morph *D. suzukii* but was no less effective in reducing mortality after 72 h [24]. Regardless, there is some evidence that insecticides combined with *S. cerevisiae* results in faster mortality (2–6 h) in comparison to commercially available protein-based baits [50].

Most previous studies have focused on the yeasts *S. cerevisiae* and *H. uvarum* [22,23,24,25,27]. However, there is a range of attractive yeast species and combinations that remain untested as phagostimulants for *D. suzukii* [34,36]. Given the observation that attractiveness varies between single yeast species and combinations of yeast species [36], it may be the case that blends of yeasts can be optimised for attraction to *D. suzukii*. Here, we investigate the effectiveness of single and combinations of co-fermented yeast species combined with insecticides in laboratory assays to evaluate their effectiveness as phagostimulant baits for use in Integrated Pest Management strategies to control *D. suzukii*. Specifically, the following hypotheses are tested: (1) combinations of co-fermented attractive yeasts will be more effective baits than single species, and (2) the effectiveness of yeast baits will differ between winter- and summer-morph *D. suzukii*.

## 2. Materials and Methods

### 2.1. Drosophila Cultures

An Italian strain of *D. suzukii* derived from flies collected in 2013 near Trento, Italy was used which was not exposed to insecticide since its establishment in the laboratory. Summer-morph flies were housed in BugDorm cages (32.5 × 32.5 × 32.5 cm) (MegaView Science Co., Ltd., Taiwan) at 89% humidity provided by damp blue absorbent paper on the roof and base of the cages at 22 ± 1.5 °C with a 16:8 h light: dark photoperiod [45]. *Drosophila* Quick Mix Medium blue (Blades Biological Ltd., Cowden, UK) sprinkled with *S. cerevisiae* (dried baker’s yeast) was used to rear summer-morph flies [36]. Additionally, cages were provisioned with frozen raspberries, weekly [45]. To generate winter-morph *D. suzukii* for the experiments, summer-morph adult flies were transferred from culture cages to square or circle-based Drosophila Bottles (177 mL, Fisherbrand) filled with 50 mL cornmeal media (1% agar, 9% sugar, 9% pre-cooked ground maize, 2% baker’s yeast, 5% malt, 1% soy flour, 0.3% propionic acid, and 0.3% methyl 4-hydroxybenzoate pre-dissolved in 70% ethanol). Flies were left to oviposit and larvae to develop for seven days whereupon adult flies were removed and the bottles maintained at 10 °C, 00:24 h light: dark. Before use in experiments, winter-morph adult *D. suzukii* were transferred to *Drosophila* bottles containing 50 mL of the *Drosophila* Quick Mix Medium sprinkled with yeast and were then acclimatised to 22 °C and 16:8 h light: dark photoperiod over a three-day period.

### 2.2. Yeast Cultures

Yeast species were from the Goddard culture collection at University of Lincoln (see Supplementary Material Table S1 for details on origin). All yeasts were grown at 30 °C with 120 rpm shaking. Yeasts were pre-cultured for 24 h in yeast peptone dextrose media (YPD; 1% yeast extract, 2% peptone, and 2% dextrose) whereupon optical density (600 nm) was assessed and 1 × 10^6^ yeast cells per mL were transferred to new YPD media and cultured for 48 h (N = 1 per yeast treatment). Yeast cells were grown (fermented) either alone or co-fermented. Where yeasts were co-fermented, cultures were inoculated with equal numbers of cells (totalling 1 × 10^6^ cells per mL) from each yeast species. For yeast baits, *H. uvarum*, *C. zemplininia* and co-fermented *H. uvarum* + *C. zemplininia* were tested for both summer- and winter-morphs, in addition to *M. pulcherrima* and co-fermented *M. pulcherrima + H. uvarum* for summer-morphs (Table 1). Five yeast treatments were tested alongside water positive and negative controls, YPD positive and negative media controls and commercially available Combi-protec (5% *v*/*v* solution) positive control [47], for summer-morph *D. suzukii*. A reduced number of treatments was tested for winter-morphs due to limitations in fly numbers (Table 1). All treatments were prepared on the day of use and mixed directly with either sterile water for the negative controls or an insecticide at the requisite concentration, shown in previous studies to be a discriminatory concentration and not to kill all adult *D. suzukii* [24].

### 2.3. Laboratory Jar Bioassay

Jar-bioassays were set up to determine the effect of combining different yeast treatments (single yeasts and combinations), with three separate insecticides; spinosad (Tracer, Dow AgroSciences, Zionsville, IA, USA), cyantraniliprole (Exirel, DuPont, Wilmington, DE, USA) and lambda-cyhalothrin (Hallmark Zeon, Syngenta, Basel, Switzerland) [24]. Insecticides were added at concentrations that ensured not all flies were killed 3.6 mg l^−1^, 18.9 and 3.8 for spinosad (Tracer), cyantraniliprole (Exirel) and lambda-cyhalothrin (Hallmark Zeon), respectively [24]. These concentrations were determined in preliminary range finding tests by [24] using the same laboratory reared *D. suzukii* population.

Jars (750 mL clear plastic jars; 103 mm diameter, 95 mm height, Involvement Packaging Ltd.) modified with a fine mesh covered ventilation hole (10 mm diameter), with damp filter paper (90 mm, Fisherbrand) on the base were used [24]. Conditions inside the jar were on average 22.4 °C and 92.7% humidity. Filter paper was re-wetted with 500 μL distilled water as required. Each jar (N = 5 replicates per treatment) contained three similar sized (approximately 30 × 20 mm) wild blackberry (*Rubus* species) leaves unsprayed with pesticide, picked the day before the experiment and stored at 2 °C. Six × 10 μL droplets of treatment or control solution (three on the upper surface, each side of the mid-vein) were applied per leaf [24].

Each jar contained leaves with either a bait combined with insecticide or an insecticide treatment only (positive control) (Table 1). The treatments or controls were applied to two of the three leaves with the third leaf receiving six droplets of sugar water as a food source (160 g l^−1^, 16%) [24]. Leaves were left to dry in a fume hood for 1–2 h prior to use and arranged with the insecticide or control leaves on one side of the jar and the sugar leaf on the opposite side (Figure S1). A 35 mm Petri dish containing grape juice agar (34.7 g Agar, 333 mL red grape juice, 33.3 g dextrose and 2.0 g Nipagin per litre distilled water) was also placed in each jar [24].

Twelve *D. suzukii* (eight females and four males) between 3–10 days old were added to each replicate jar (N = 5 replicates per treatment). Flies were anaesthetised using CO_2_, sex determined then starved for seven hours prior to the experiment starting, whereupon they were briefly anaesthetised with CO_2_ before being inserted into the jars in the space between the leaves (Figure S1). Adult fly mortality was recorded at 1, 2, 4, 8, 24, 32 and 48 h. Flies which were heavily moribund (defined as individuals clearly close to death, on their back or sides with one or more legs twitching) were classified as dead.

### 2.4. Statistical Analysis

Differences in mortality were analysed using parametric survival regression analysis. Since different concentrations of each insecticide were used, treatment effects on mortality were analysed using separate parametric survival regressions, the significance of which was assayed using ANOVA following model simplification as per [53]. Data from the four treatments, common to both morphological types (*C. zemplininia*, *H. uvarum* and *H. uvarum* + *C. zemplininia* and YPD media negative control; Table 1) was also analysed to assess the effect of morph on mortality, separately for each insecticide. All pairwise comparisons were done using Benjamini-Hochberg corrected Log-Rank tests.

Probit analyses (two factor model) was used to identify the LT_50_ (time to 50% population mortality) for each bait treatments and insecticide separately. Statistical analyses were carried out in R version 4.0.2 [54] and the ‘survival’ package [55] was used for the separate parametric survival regression with the ‘survminer’ package [56] for the multiple comparisons. The package ‘lme4′ [57] was used for linear regression and ‘emmeans’ [58] for multiple comparisons. The ‘drc’ package was used for probit analysis [59].

## 3. Results

### 3.1. Summer-Morph Mortality of D. suzukii

For all three insecticides (spinosad, lambda-cyhalothrin, and cyantraniliprole) treatment had a significant effect on summer-morph *D. suzukii* mortality (Parametric survival regression Δ deviance = 222.27, df = 9, *p* < 0.001; Δ deviance = 111.14, df = 9, *p* < 0.001; Δ deviance = 256.22, df = 9, *p* < 0.001, respectively).

**Figure 1:**
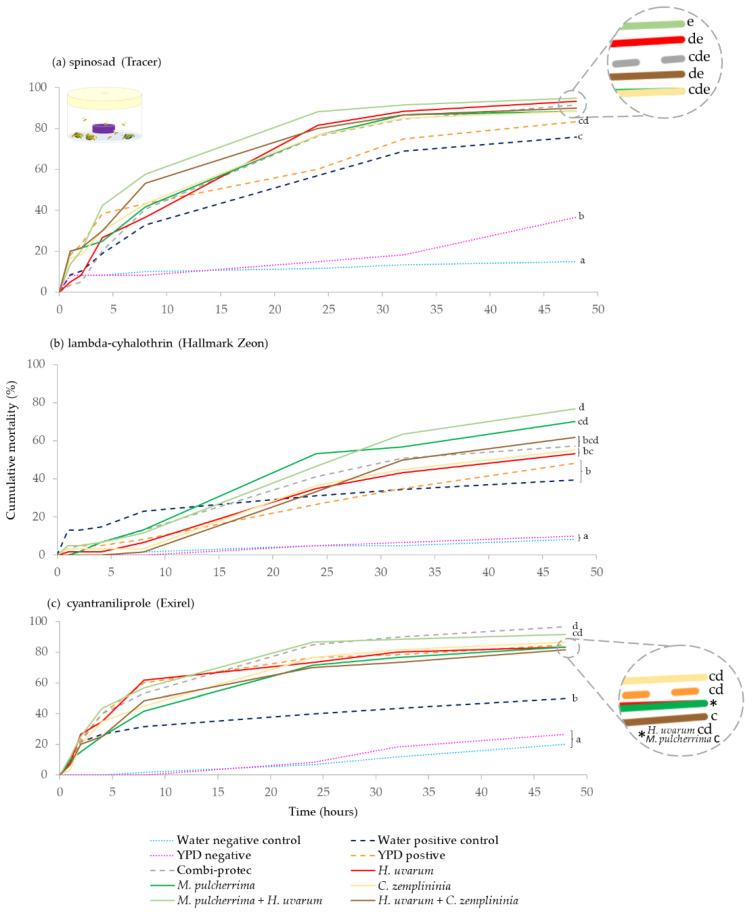
Cumulative percentage mortality of summer-morph *D. suzukii* exposed to yeast phagostimulant baits (*H. uvarum*, *M. pulcherrima*, *C. zemplininia*, *M. pulcherrima* + *H. uvarum* and *H. uvarum* + *C. zemplininia*) in combination with insecticides (**a**) spinosad, (**b**) lambda-cyhalothrin or (**c**) cyantraniliprole compared to Combi-protec, YPD media and water positive controls (dashed lines) and YPD and water negative controls (dotted lines). Experiments lasted 48 h, and the conditions inside the jars were 22.4 °C and 92.7% humidity with 16:8 h light: dark photoperiod. Separate log-Rank Pairwise comparisons for each insecticide were used to determine significance between treatment and different letters at the ends for lines denote significance differences (within each insecticide, *p* < 0.05).

For spinosad, all insecticide treatments (baits and positive controls) caused significantly greater *D. suzukii* mortality than both the YPD media and water negative controls (*p* < 0.001). The yeast baits, *H. uvarum*, *H. uvarum* + *C. zemplininia* and *M. pulcherrima + H. uvarum* in combination with spinosad, caused significantly greater mortality than the water (spinosad) positive control (*p* = 0.038, *p* = 0.017 and *p* < 0.001, respectively). Additionally, *M. pulcherrima* + *H. uvarum* with spinosad caused significantly greater *D. suzukii* mortality than the YPD media positive control (*p* = 0.040). There was also higher mortality in the YPD negative control than the water negative control (*p* = 0.030) (Figure 1a).

All lambda-cyhalothrin treatments caused significantly greater *D. suzukii* mortality than both the YPD media and water only negative controls (*p* < 0.001). *M. pulcherrima*, and *M. pulcherrima + H. uvarum* in combination with lambda-cyhalothrin produced significantly higher mortality than the water positive (lambda-cyhalothrin) control (*p* = 0.021, *p* = 0.003, respectively) and YPD positive control (*p* = 0.024, *p* = 0.003, respectively). Additionally, *M. pulcherrima + H. uvarum* with lambda-cyhalothrin resulted in greater mortality than *C. zemplininia* and *H. uvarum*, with lambda-cyhalothrin (*p* = 0.024 and *p* = 0.021) (Figure 1b).

All cyantraniliprole treatments resulted in greater mortality than both the YPD media and water negative controls (*p*-values ranging from < 0.001 to 0.003). All bait treatments with cyantraniliprole, including YPD positive control, had higher *D. suzukii* mortality than the water positive control (*p* ranging from < 0.001 to 0.003). Additionally, Combi-protec with cyantraniliprole caused greater mortality than *H. uvarum* + *C. zemplininia* and *M. pulcherrima*, with cyantraniliprole (*p* = 0.045 and *p* = 0.046) (Figure 1c).

### 3.2. Winter-Morph Mortality of D. suzukii

A subset of baits (*H. uvarum*, *C. zemplininia*, *H. uvarum* + *C. zemplininia* and YPD media negative control; Table 1) were tested against the winter-morph. As with *D. suzukii* summer-morphs, bait treatments in combination with an insecticide; spinosad, lambda-cyhalothrin, or cyantraniliprole, had a significant effect on winter-morph mortality (Parametric survival regression Δ deviance = 173.96, df = 3, *p* < 0.001; Δ deviance = 166.90, df = 3, *p* < 0.001; Δ deviance = 229.80, df = 3, *p* < 0.001, respectively).

**Figure 2:**
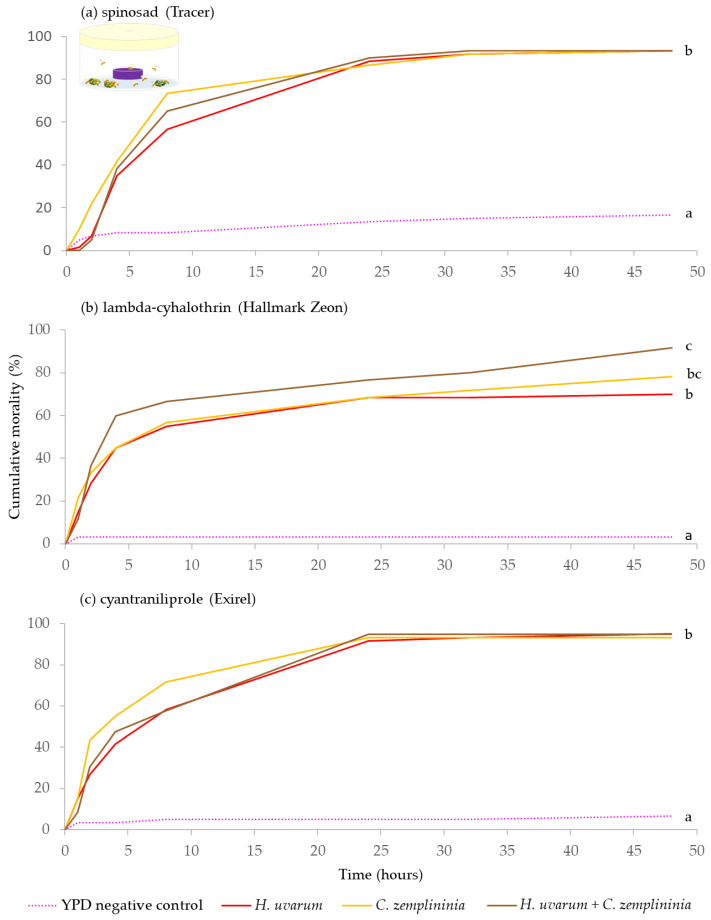
Cumulative percentage mortality of winter-morph *D. suzukii* exposed to yeast phagostimulant baits (*H. uvarum*, *C. zemplininia* and *H. uvarum* + *C. zemplininia*) in combination with the insecticides (**a**) spinosad, (**b**) lambda-cyhalothrin or (**c**) cyantraniliprole, compared to YPD media (negative control, dotted line). Experiments lasted 48 h, conditions inside the jars were 22.4 °C and 92.7% humidity with 16:8 h light: dark photoperiod. Separate log-Rank Pairwise comparisons for each insecticide were used to determine significance between treatment and different letters at the ends of lines denote significance differences (within each insecticide, *p* < 0.05).

Across the three insecticide experiments (spinosad, lambda-cyhalothrin or cyantraniliprole), when combined with *C*. *zemplininia*, *H. uvarum,* or *H. uvarum* + *C. zemplininia* caused significantly greater mortality than the YPD media negative control (*p* < 0.001). Additionally, *H. uvarum* + *C. zemplininia* paired with lambda-cyhalothrin caused significantly greater mortality than *H. uvarum* alone combined with lambda-cyhalothrin (*p* = 0.033) (Figure 2b).

### 3.3. Differences in Mortality between D. suzukii Winter- and Summer-Morphs

There was a significant interaction between treatment and morph for all three insecticides (Parametric survival regression Δ deviance = 10.81, df = 3, *p* = 0.013; Δ deviance = 10.18, df = 3, *p* = 0.017; Δ deviance = 19.17, df = 3, *p* < 0.001, spinosad, lambda-cyhalothrin and cyantraniliprole, respectively) showing that *D. suzukii* morphs differentially responded to the treatments.

**Figure 3:**
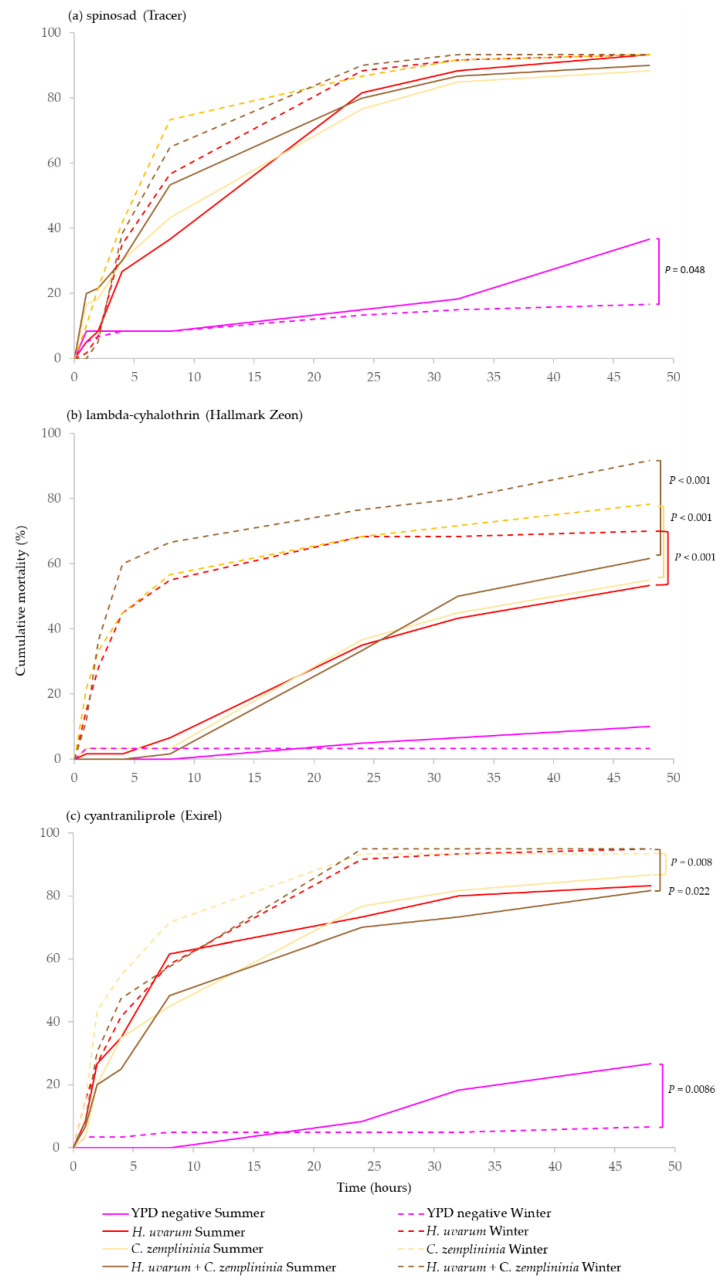
Comparison of cumulative percentage mortality of summer- (solid lines) and winter-morph (broken lines) *D. suzukii* of yeast phagostimulant baits (*H. uvarum*, *C. zemplininia* and *H. uvarum* + *C. zemplininia*) in combination with the insecticides (**a**) spinosad, (**b**) lambda-cyhalothrin or (**c**) cyantraniliprole, compared to YPD media (negative control). Experiments lasted 48 h, conditions inside the jars were 22.4 °C and 92.7% humidity with 16:8 h light: dark photoperiod. Separate log-Rank Pairwise comparisons for each insecticide were used to determine significance in mortality between morphological type of *D. suzukii* and coloured bars with *p*-values connecting lines denote any significance difference in mortality between *D. suzukii* summer- and winter-morphs for the different insecticides (*p* < 0.05).

*C. zemplininia* combined with lambda-cyhalothrin or cyantraniliprole but not spinosad, caused significantly greater mortality (between 8–23%) in winter- than in summer-morphs (*p* < 0.001, 0.008 and *p* = 0.053, respectively). *H. uvarum* + *C. zemplininia* combined with lambda-cyhalothrin or cyantraniliprole caused significantly greater mortality (13–30%) in winter-morph flies (*p* < 0.001 and *p* = 0.022) and *H. uvarum* only, combined with lambda-cyhalothrin also caused significantly greater mortality (17%) to winter-morph flies (*p* < 0.001). Additionally, there was marginally significantly greater mortality (20%) of summer-morphs (*p* = 0.048) in the YPD media negative control treatments for spinosad and cyantraniliprole experiments (*p* = 0.048, *p* = 0.009) but not lambda-cyhalothrin (Figure 3).

### 3.4. Median Lethal Time (Time until Death) of 50% (LT_50_) of Summer-Morph D. suzukii to Insecticides Combined with Phagostimulant Baits

**Figure 4:**
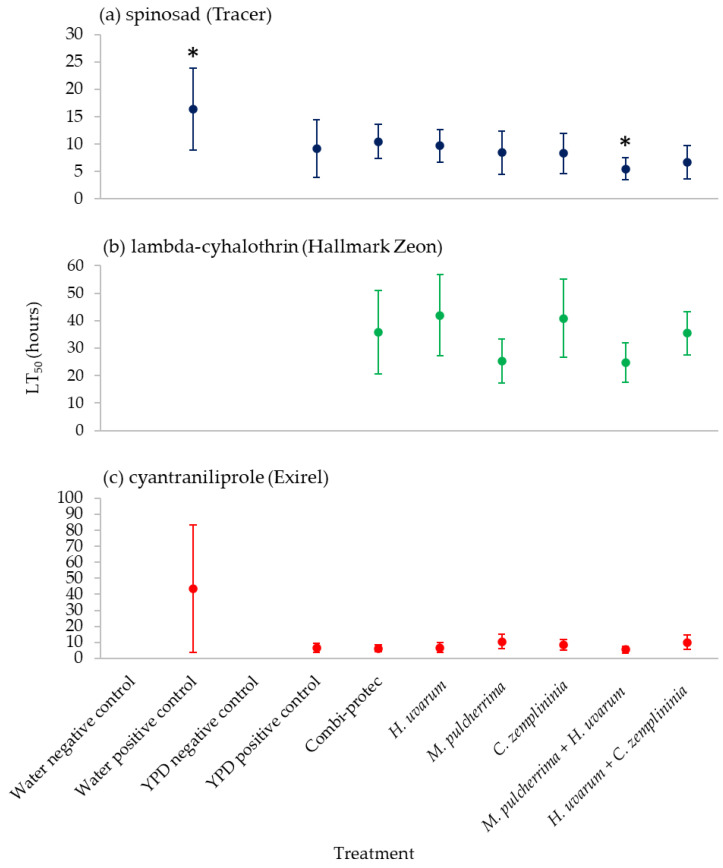
Median Lethal Time (time until death) of 50% (LT_50_) of summer-morph *D. suzukii* in hours of yeast phagostimulant baits (*H. uvarum*, *M. pulcherrima*, *C. zemplininia*, *M. pulcherrima* + *H. uvarum* and *H. uvarum* + *C. zemplininia*) in in combination with the insecticides (**a**) spinosad, (**b**) lambda-cyhalothrin or (**c**) cyantraniliprole compared to Combi-protec, YPD media and water positive controls and YPD and water negative controls. * Treatments significantly different where 95% confidence intervals (horizontal error bars) do not overlap. Treatments (water and media controls for lambda-cyhalothrin) that did not reach 50% mortality by the end of the experiment are omitted from graphs.

*M. pulcherrima* + *H. uvarum* with spinosad was the only phagostimulant bait, including the commercial product (Combi-protec), that significantly reduced the time-to-kill of 50% of the *D. suzukii* population (LT_50_) compared to the water positive control (Figure 4; Table S2). Lambda-cyhalothrin combined with water only did not reach 50% mortality by the end of the experiment, although combining the insecticide with bait did reduce the numbers of *D. suzukii* by at least 50% by the end of the experiment (Figure 4; Table S2). Only summer-morph *D. suzukii* data was analysed due to winter-morphs not being tested against water positive control (Table 1).

## 4. Discussion

Yeasts are effective phagostimulant baits that can be combined with insecticides to reduce the dose required, by up to 96%, while retaining comparable levels of *D. suzukii* control [26]. However, little is known about how combining yeasts affects the efficacy of these baits and whether effectiveness will vary depending on morphological type of *D. suzukii*. This study tested the hypotheses that combinations of co-fermented attractive yeasts are more effective as baits than single yeast species and their effectiveness against winter- and summer-morph *D. suzukii* will differ, finding some evidence to support both hypotheses.

Yeast phagostimulant baits applied to foliage (blackberry leaves) generally increased the mortality of *D. suzukii* compared to exposure to the same dose of insecticide combined with water only. Insecticidal efficacy was improved by the addition of certain yeast-based phagostimulants. *H. uvarum*, *H. uvarum* + *C. zemplininia* or *M. pulcherrima + H. uvarum* in combination with spinosad, *M. pulcherrima*, and *M. pulcherrima* + *H. uvarum* with lambda-cyhalothrin, and all yeast baits combined with cyantraniliprole caused significantly greater mortality than insecticide and water alone (Figure 1). The findings here agree with previous studies which show *H. uvarum* is an effective phagostimulant bait [22,24,25,26,27]. *H. uvarum* combined with spinosad, cyantraniliprole or lambda-cyhalothrin increased *D. suzukii* mortality compared to exposure to an insecticide alone [22,24]. We report a similar trend for spinosad and cyantraniliprole but not lambda-cyhalothrin: this discrepancy may be attributed to differences in length of exposure to insecticides and/or differences in strain of *H. uvarum* or yeast culture media used, both of which may affect *D. suzukii* attraction to yeast [35,36].

There was some evidence that phagostimulant baits resulted in an increased rate of mortality against summer-morph *D. suzukii* (approximately 1.5 and eight times) when combined with spinosad or cyantraniliprole, compared to insecticides with just water (Figure 4; Table S2). Despite this, only *M. pulcherrima* + *H. uvarum* combined with spinosad had a significantly lower LT_50_ (threefold) than the water positive control (Figure 4; Table S2). After a short exposure time (two hours) *S. cerevisiae* + sugar baits combined with spinosad caused greater mortality than insecticide applied alone [50]. Faster kill rates are desirable as this potentially limits the number of eggs laid by female *D. suzukii* thus potentially reducing fruit damage. We only present data on speed of mortality of phagostimulant baits compared to insecticide and water alone for summer-morphs but recommend that the efficacy of these baits should also be assessed for winter-morphs.

Only *M. pulcherrima + H. uvarum* combined with spinosad or lambda-cyhalothrin and *M. pulcherrima* with lambda-cyhalothrin caused significantly higher mortality than YPD media combined with the respective insecticide (Figure 1). *H. uvarum* combined separately with the three insecticides did not cause significantly greater mortality than YPD and insecticides (Figure 1) possibly due to YPD being attractive [33]. Additionally, YPD media combined with cyantraniliprole, but not the other insecticides, caused significantly greater mortality than insecticide alone (35% more after 48 h; Figure 1). This finding suggests that in certain cases YPD, a relatively cheap culture media, may be an effective phagostimulant worthy of further investigation.

There was limited evidence supporting the hypothesis that combinations of yeasts are more effective than single species. *M. pulcherrima* + *H. uvarum,* combined with lambda-cyhalothrin, resulted in higher summer-morph *D. suzukii* mortality than *C. zemplininia* (22% higher) or *H. uvarum* for both summer- and winter-morph flies (23% and 22%, Figure 1 and Figure 2). *H. uvarum* is an effective yeast phagostimulant [22,24,25,26,27] but efficacy might be further promoted by mixing with other yeast isolates, e.g., *M. pulcherrima,* at least when combined with certain insecticides (Figure 1). There was no evidence that *M. pulcherrima* + *H. uvarum* co-fermented in YPD improved attraction compared to *H. uvarum* alone [33]. However, attraction to a bait does not necessarily increase its potential as a phagostimulant when combined with an insecticide [24]. In this study live yeast cells were combined with insecticides on blackberry leaves and could have potentially further interacted with each other, as well as with the epiphytic leaf microbes, which could have influenced attraction. Previous work has shown that interactions during co-cultures of yeast and bacteria modulated attraction of *D. melanogaster* [44].

There was no conclusive evidence that yeast baits were more effective phagostimulants than a current commercial bait (Combi-protec) to summer-morph *D. suzukii*, which is consistent with previous findings [24,26]. Additionally, Combi-protec was the most effective bait tested when combined with cyantraniliprole [26] and caused significantly higher mortality of summer-morph *D. suzukii* compared to *M. pulcherrima* and *H. uvarum* + *C. zemplininia* (13–15% higher, Figure 1). However, there is some evidence with other yeast species (*S. cerevisiae*) and different commercially available protein-based baits (NuLure and GF-120), that yeasts increased mortality, at least in the short term (2–6 h) compared to protein-based baits [50].

Most previous studies investigating yeasts as phagostimulants have focused on *S. cerevisiae* and *H. uvarum*, e.g., [22,23,24,25,26,27], although *M. pulcherrima* and *Cryptococcus tephrensis* have also been tested [23]. Whilst *S. cerevisiae* and *H. uvarum* are undoubtably effective phagostimulants, this study has expanded the range of *D. suzukii* bait possibilities for future attract-and-kill strategies. These experiments used a laboratory strain of *D. suzukii* which has not been exposed to insecticides since establishment in the laboratory in 2014. As resistance of *D. suzukii* to spinosad has been recorded in field populations in the USA [19], it is also important to assess the effectiveness of potential phagostimulant baits in combination with insecticides to field populations.

Finally, it was hypothesised that the effectiveness of yeast phagostimulant baits would differ between winter- and summer-morph *D. suzukii.* There was evidence to support this as all yeasts and their combinations combined with lambda-cyhalothrin caused significantly (17–30%) higher mortality in winter-morphs than summer-morphs. This was also apparent for *C. zemplininia* or *H. uvarum* + *C. zemplininia* combined with cyantraniliprole, but to a lesser extent (7–13%, Figure 3). These differences could be the result of detection and attraction differences to yeasts between the different morphs. For example, *C. zemplininia* was more attractive to winter- than summer-morph females although co-fermented *H. uvarum* + *C. zemplininia* and *H. uvarum* alone were not [33]. The differences in mortality between morphs for yeast baits were more pronounced in combination with lambda-cyhalothrin than other insecticides (Figure 3). Certain pyrethroids, cyhalothrin and cyhalothrin but not deltamethrin, are somewhat repellent to summer-morph *D. suzukii* at low concentrations [27,60] and olfactory responses differ between *D. suzukii* winter- and summer-morphs [29]. For example, summer-morphs are significantly repelled by geosmin whereas winter-morphs are attracted (although not significantly) [30]. It is conceivable that winter-morph *D. suzukii* are less able to detect pyrethroids, less repelled by them, and/or more toxicologically sensitive.

Conversely, winter-morphs were previously found to be less sensitive to insecticides than summer-morph *D. suzukii* compared to this study [24]. However, both temperature and photoperiod conditions varied between the morphs (21 °C and 16: 8 light: dark; 13 °C 8: 16 light: dark for summer- and winter-morphs, respectively) [24]. Our winter-morph experiments were conducted at a higher temperature and with a longer photoperiod, presumably increasing the flies’ activity and improving exposure which could explain this discrepancy. To clarify, experiments carried out for both morphs at both conditions would be needed. The effectiveness of phagostimulants against the winter-morph should be tested in field situations to ensure efficacy is realised in realistic conditions [36]. Differences in the effectiveness of yeast baits to summer- and winter-morphs is an important finding suggesting that phagostimulant baits might be tailored to better target the two morphological stages as part of Integrated Pest Management control strategies. Winter-morph females make up the majority of the flies entering the crop at the start of the growing season [9], making it advantageous to reduce the numbers of overwintering flies and/or winter-morphs early in the season. Further work is needed to test if current commercial baits are as effective for both morphs in a commercial setting.

## 5. Conclusions

Yeasts are important candidates as phagostimulant baits in combination with insecticides for attract-and-kill strategies for *D. suzukii* control. We have identified yeast species and combinations which are potentially effective phagostimulant baits. Additionally, in some cases combinations are more effective phagostimulant baits than single yeast species. We also show that effectiveness of yeast phagostimulants can vary between *D. suzukii* morphs, suggesting there is potential to tailor baits according to seasonality. These findings contribute to developing sustainable lower insecticide inputs into horticulture management controls for both morphological stages of *D. suzukii* and likely reduce the impact of insecticides on beneficial insects like pollinators and natural enemies.

## Figures and Tables

**Table 1:** Single and fermented combinations of yeasts tested as baits with winter- and summer-morph *Drosophila suzukii*. Yeasts were compared to water or yeast peptone dextrose media (YPD; 1% yeast extract, 2% peptone, and 2% dextrose) controls with (positive) or without (negative) insecticides. A commercial product Combi-protec was included as a second positive control for summer-morphs experiments. X denotes which treatments were included in each experiment.

Treatment	Positive/Negative Control	Summer-Morph Experiments	Winter-Morph Experiments
*Hanseniaspora uvarum*	-	X	X
*Metschnikowia pulcherrima*	-	X	
*Candida zemplininia*	-	X	X
*M. pulcherrima* + *H. uvarum*	-	X	
*H. uvarum* + *C. zemplininia*	-	X	X
Water	Negative	X	
Water	Positive	X	
YPD media	Negative	X	X
YPD media	Positive	X	
Combi-protec	Positive	X	

## Data Availability

All raw data included in this study can be found in the supplementary material.

## Supplementary Materials

The following supporting information can be downloaded at: https://www.mdpi.com/article/10.3390/insects13110995/s1, Figure S1. Jar-bioassay set up: Figure S2. Mean eggs laid by summer-morph *D. suzukii*: Table S1: Origin of yeast isolates: File S1 data; Table S2. Median Lethal Time (LT_50_) of summer-morph *D. suzukii*.
