# Supplementary material for: The Efficacy of Yeast Phagostimulant Baits in Attract-and-Kill Strategies Varies between Summer- and Winter-Morphs of Drosophila suzukii
Source: Insects. 2022 Oct 29;13(11):995. doi: 10.3390/insects13110995 (PMC9696471; doi:10.3390/insects13110995)
Supplement: Supplementary file 1 [file insects-13-00995-s001.zip › Supplementary material. Jones et al. The efficacy of yeast phagostimulant baits 5.9.22.pdf]

Supplementary material: The efficacy of yeast phagostimulant baits in attract-and-kill strategies varied against summer- and winter-morphs of *Drosophila suzukii*

Rory Jones [1,2,3,*], Paul E. Eady [1], Matthew R. Goddard [1,4] and Michelle T. Fountain [2]

[1] School of Life Sciences, University of Lincoln, Lincoln, LN6 7DL, UK
[2] NIAB, East Malling, Kent ME19 6BJ, UK
[3] Current address: ADAS, Boxworth, Cambridge CB23 4NN
[4] The School of Biological Sciences, The University of Auckland, Auckland, New Zealand * Correspondence: r.jones26@live.co.uk

Table S1: Origin, source and strain of yeast isolates used in this study.

**Species**	**Strain**	**Origin**	**Source**	**Reference**
Hanseniaspora uvarum	201	New Zealand	Chardonnay fruit	Gayevskiy and Goddard, 2012. ISME Journal 6:1281-90
Metschnikowia pulcherrima	190	New Zealand	Sauvignon Blanc ferment	Goddard culture collection
Candida zemplininia	164	New Zealand	Chardonnay ferment	Anfang et al., 2009. Australian Journal of Grape and Wine Research 15:1-8

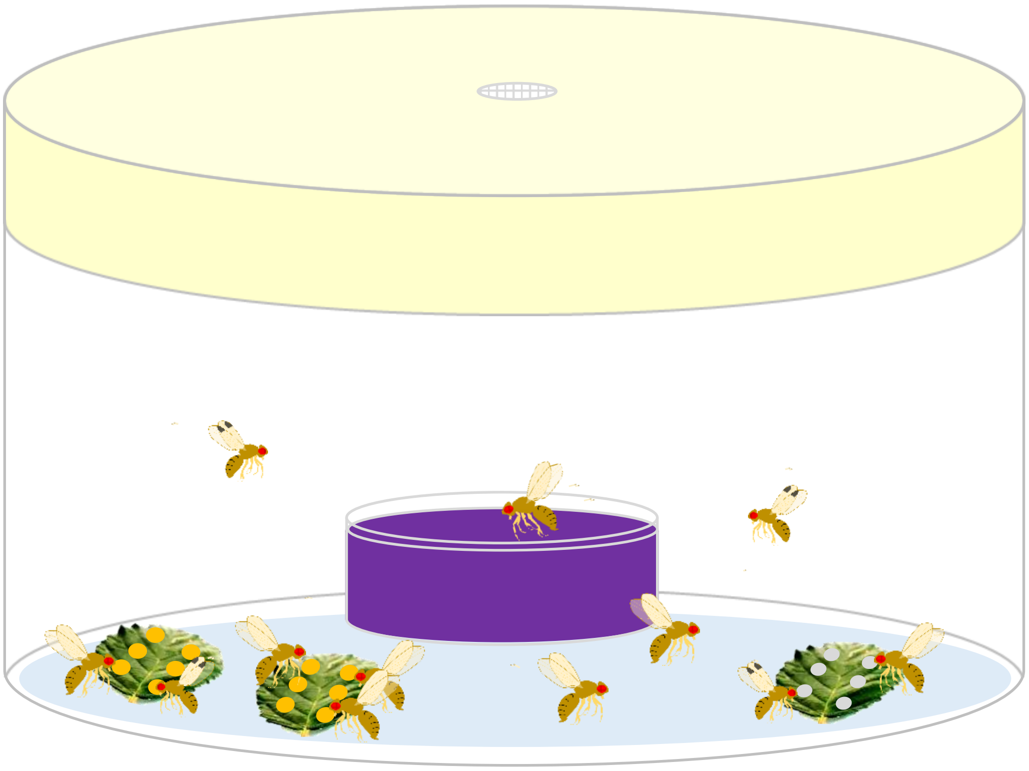

Figure S1. Jar-bioassay set up following [21]. Jars (750 mL clear plastic jars; 103 mm diameter, 95 mm height, Involvement Packaging Ltd.) modified with a fine mesh covered ventilation hole (10 mm diameter), with damp filter paper (90 mm, Fisherbrand) on the base. Each jar contained three similar sized (approximately 30 × 20 mm) blackberry leaves with 6 x 10 µL droplets per leaf (three on each side); two leaves had insecticide or control and bait or control and the other leaf sugar solution (160 g l^{-1}, 16%). 35 mm petri dish (Corning) containing grape juice agar for egg laying were also placed in each jar. Approximately 12 flies (eight females and four males) between 3-10 days old were anaesthetised briefly using CO_2 and inserted into the jars in the space between leaves (N=5 replicates per treatment).

Table S2. Median Lethal Time (time until death) of 50% (LT_{50}) of summer-morph *D. suzukii* in hours of yeast phagostimulant baits (*H. uvarum*, *M. pulcherrima*, *C. zemplininia*, *M. pulcherrima* + *H. uvarum* and *H. uvarum* + *C. zemplininia*) in combination with insecticides (spinosad, lambda-cyhalothrin and cyantraniliprole) compared to Combi-protec, YPD media and water positive controls, and YPD and water negative controls. * Treatments that did not reach 50% mortality by the end of the experiment (48 hours).

		LT_{50} (hours)	95% confidence intervals	
Insecticide	Treatment	Estimate (± SE)	Lower	Upper
spinosad	Water negative control	*	-	-
	Water positive control	16.35 (± 3.82)	8.86	23.85
	YPD negative control	*	-	-
	YPD positive control	9.17 (± 2.69)	3.89	14.45
	Combi-protec	10.47 (± 1.61)	7.31	13.64
	H. uvarum	9.71 (± 1.52)	6.73	12.69
	M. pulcherrima	8.45 (± 2.02)	4.50	12.41
	C. zemplininia	8.31 (± 1.88)	4.62	12.00
	M. pulcherrima + *H. uvarum*	5.50 (± 1.03)	3.48	7.52
	H. uvarum + *C. zemplininia*	6.72 (± 1.56)	3.67	9.78
lambda-cyhalothrin	Water negative control	*	-	-
	Water positive control	*	-	-
	YPD negative control	*	-	-
	YPD positive control	*	-	-
	Combi-protec	35.86 (± 7.75)	20.67	51.05
	H. uvarum	41.94 (± 7.53)	27.18	56.71
	M. pulcherrima	25.26 (± 4.04)	17.34	33.18
	C. zemplininia	40.87 (± 7.29)	26.57	55.17
	M. pulcherrima + *H. uvarum*	24.69 (± 3.66)	17.52	31.85
	H. uvarum + *C. zemplininia*	35.41 (± 4.04)	27.49	43.33
cyantraniliprole	Water negative control	*	-	-
	Water positive control	43.52 (± 20.27)	3.79	83.24
	YPD negative control	*	-	-
	YPD positive control	6.47 (± 1.48)	3.56	9.38
	Combi-protec	6.17 (± 1.11)	4.00	8.34
	H. uvarum	6.79 (± 1.58)	3.70	9.89
	M. pulcherrima	10.55 (± 2.31)	6.02	15.08
	C. zemplininia	8.49 (± 1.72)	5.12	11.86
	M. pulcherrima + *H. uvarum*	5.52 (± 1.11)	3.35	7.69
	H. uvarum + *C. zemplininia*	9.97 (± 2.26)	5.53	14.41

Egg-laying

Numbers of eggs laid in the grape juice agar was recorded at 48h. Eggs were counted under a microscope at x50 magnification. Eggs laid in grape juice agar were analysed using a generalised linear model with a Poisson error structure and Firth's adjustment. Treatment and insecticide were treated as fixed factors. Significance of the model was analysed using ANOVA, to account for overdispersion in the model the deviances were divided by the overdispersion parameter.

No Egg-laying was recorded for winter-morph experiments and egg-laying was inconsistent across summer-morph experiments. Overall, there was a significant effect of treatment, insecticide and their interaction on summer-morph *D. suzukii* oviposition (treatment Δ deviance = 904.26, df = 9, $P < 0.001$, insecticide Δ deviance = 868.55, df = 2, P = 0.027; treatment*insecticide Δ deviance = 615.63, df = 18, $P < 0.001$, respectively). *C. zemplininia* combined with spinosad resulted in significantly fewer eggs laid in the grape juice agar than *H. uvarum* with spinosad (P = 0.048). There were no significant differences between the numbers of eggs laid between treatments in the lambda-cyhalothrin experiment. Flies exposed to cyantraniliprole with *M. pulcherrima* + *H. uvarum* oviposited significantly fewer eggs compared to the YPD negative control (P = 0.020) (supplementary material, Fig. S2). No oviposition occurred in the winter-morph experiments [21].

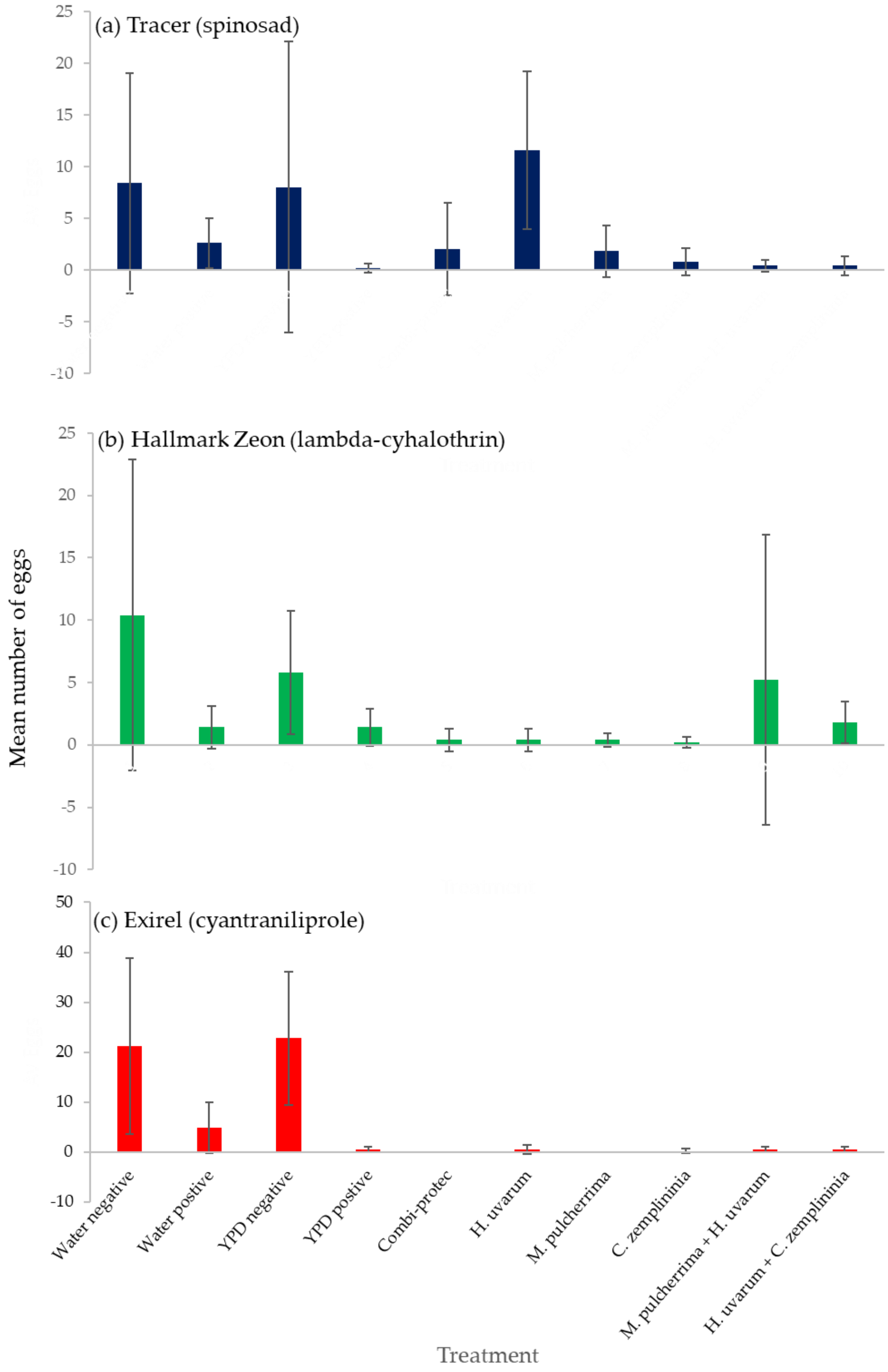

Figure S2. Mean (± SE) eggs laid in grape juice ager by summer-morph *D. suzukii* after 48 hours from the various yeast phagostimulant baits alongside Combi-protec, YPD media and water positive controls and YPD and water negative controls combined with three separate insecticides (a) spinosad, (b) lambda-cyhalothrin and (c) cyantraniliprole.
